# Clusters of risk for the occurrence of leprosy and disabilities in children under 15 years of age in Cuiabá: a geospatial study

**DOI:** 10.1590/1980-549720230006.2

**Published:** 2023-01-09

**Authors:** José Francisco Martoreli, Antônio Carlos Vieira Ramos, Thaís Zamboni Berra, Murilo César do Nascimento, Reginaldo Bazon Vaz Tavares, Heriederson Sávio Dias Moura, Débora Falleiros de Mello, Josilene Dália Alves, Ricardo Alexandre Arcêncio

**Affiliations:** I Universidade de São Paulo, Escola de Enfermagem de Ribeirão Preto, Escola de Enfermagem – Ribeirão Preto (SP), Brasil.; II Universidade Federal de Alfenas, Campus Sede – Alfenas (MG), Brasil.; III Universidade Federal de Mato Grosso – Cuiabá (MT), Brasil.

**Keywords:** Leprosy, Child health, Social vulnerability, Spatial analysis, Hanseníase, Saúde da criança, Vulnerabilidade social, Análise espacial

## Abstract

**Objective:**

This study aimed to analyze the spatial distribution of leprosy and disabilities in children under 15 years of age in Cuiabá.

**Methods:**

Ecological study carried out in the city of Cuiabá, Mato Grosso, Brazil. The study population consisted of leprosy cases in children under 15 years old notified in the Notifiable Diseases Information System, between 2008 and 2018. Based on residential addresses, cases were georeferenced. In the analysis of the spatial distribution of the cases, the estimation of the Kernel density was used and, later, the statistics of spatial, spatio-temporal and Spatial Variation in Temporal Trends were applied.

**Results:**

514 cases of leprosy were reported in children under 15 years of age in Cuiabá, with a percentage of 10.1% of cases with degree of physical disability 1 and 2.3% with degree of physical disability 2 at the time of diagnosis. With the techniques of spatial and spatio-temporal scanning, clusters of risk for leprosy were identified in the North, West, East and South regions of Cuiabá, and with the technique of Spatial Variation in Temporal Trends, a cluster was identified in the West region of Cuiabá.

**Conclusion:**

In Cuiabá, cases of leprosy in children under 15 years of age with disabilities were distributed throughout the urban area of the city, with the highest density of cases in the North and West regions, followed by the East region. The clusters with the highest Relative Risk were identified in the East and West regions, characterized by having low and medium income levels

## RESUMO

**Objetivo:**

Este estudo teve como objetivo analisar a distribuição espacial da hanseníase e as incapacidades em menores de 15 anos de idade em Cuiabá.

**Métodos:**

Estudo ecológico realizado na cidade de Cuiabá, Mato Grosso, Brasil. A população do estudo foi composta de casos de hanseníase em menores de 15 anos notificados no Sistema de Informações de Agravos de Notificação, entre os anos de 2008 e 2018. Com base nos endereços residenciais, os casos foram georreferenciados. Na análise da distribuição espacial dos casos, foi utilizada a estimativa da densidade de Kernel e, posteriormente, aplicada a estatística de varredura espacial, espaço-temporal e variação espacial nas tendências temporais.

**Resultados:**

Foram notificados 514 casos de hanseníase em menores de 15 anos em Cuiabá, com percentual de 10,1% de casos com grau de incapacidade física 1 e 2,3% com grau de incapacidade física 2 no momento do diagnóstico. Com as técnicas de varredura espacial e espaço-temporal, foram identificados aglomerados de risco para hanseníase nas regiões norte, oeste, leste e sul de Cuiabá, e com a técnica de variação espacial nas tendências temporais foi identificado um aglomerado na região oeste de Cuiabá.

**Conclusão:**

Em Cuiabá, os casos de hanseníase em menores de 15 anos com incapacidades estavam distribuídos em toda a extensão urbana da cidade, com maior densidade de casos nas regiões norte e oeste, seguida da região leste. Os aglomerados de maior risco relativo foram identificados nas regiões leste e oeste, caracterizadas por apresentar baixo e médio níveis de renda.

## INTRODUCTION

Leprosy is a chronic infectious disease with slow evolution, characterized by high infectivity and low pathogenicity, resulting in severe neuropathies, disability grading (DG), and deformities^1-3^.

Among the main international strategies and commitments of the 21^st^ century regarding diseases considered “neglected”, the Sustainable Development Goals (SDG) of the 2030 Agenda of the United Nations (UN)^4^ and the “Global Leprosy Strategy 2021– 2030 — Towards zero leprosy”, strategies that aim to eradicate the disease, reduce the number of children and adolescents diagnosed with leprosy, increase early diagnosis, and the non-occurrence of disabilities and/or deformities, in addition to combating stigma and ensuring that human rights are respected^4-6^.

The occurrence of leprosy in children under 15 years of age suggests the existence of bacilliferous household/family index cases, with strong indications of continuous and active transmission of the bacillus at home and in the community^7,8^. Often, patients and family members are unaware of any contact they have had with the disease, as well as the existence of cases of leprosy in the family or in the vicinities^9^. Often, index cases are not identified early by health services, and close and prolonged contact causes the disease to affect children and adolescents, perpetuating the disease transmission chain and making it rather difficult to be eliminated^7-11^.

Leprosy in children under 15 years of age in Brazil is a serious public health problem, with a high occurrence of cases^12^. In 2019, 27,864 new cases of leprosy were reported in Brazil, of which 1,545 (5.5%) were reported in children under 15 years of age and 2,351 (9.9%) presented DG at the time of diagnosis^13,14^.

Through a search in the literature, it was possible to identify few studies that proposed to analyze the occurrence of leprosy in children under 15 years of age in the Brazilian context. Thus, the objective of the study was to analyze the spatial distribution of leprosy and disabilities in children under 15 years of age in Cuiabá.

## METHODS

This is an ecological study^15^, using spatial and space-time analysis techniques, developed in the city of Cuiabá, capital of the state of Mato Grosso, Center-West Region of Brazil^16-18^. For the study, the 140 urban Human Development Units (HDU) in Cuiabá were considered as the ecological analysis unit^19^.

Cuiabá has a territorial area of 3,266.538 km^2^ and an estimated population of 623,614 people in 2021^17,18^. As socioeconomic indicators, the municipality has a percentage of people without schooling of 4.56% for women and 4.79% for men, Human Development Index (HDI) of 0.785 and Gini Index of 0.59. It should be noted that only 53.52% of its territory has a sewage system^17,18^.

Regarding the health system, the city has 63 Family Health Programs (FHP), distributed in four administrative regions: North, with 13 FHP and 10 Family Health Strategies (FHS) teams; South, with 21 FHP and 20 FHS; East: with 15 FHP and 10 FHS; West, with 11 FHP and 8 FHS^16^. As a study population, all new cases of leprosy in children under 15 reported in the Notifiable Diseases Information System (*Sistema de Informação de Agravos de Notificação* – SINAN) in the period from 2008 to 2018 were considered^20^.

The study data included clinical and sociodemographic information of the notified cases, such as date of notification, date of birth, gender (male and female), education, operational classification (paucibacillary (PB); multibacillary (MB)), clinical form, evaluation of the DG at the time of diagnosis (DG 0, DG 1, and DG 2), and residence address. Access to the SINAN database was obtained through authorization from the Sanitary Surveillance Service of the Regional Health Management of Cuiabá, in November 2019.

After analyzing the consistency of the database, a descriptive analysis was performed with the aim of characterizing the profile of leprosy cases in children under 15 years of age.

In the spatial analysis stage, cases of leprosy in children under 15 years of age were georeferenced based on the geographic coordinates (latitude and longitude) of the residential addresses, using the QGIS 3.14 software. For this stage, cases whose addresses were not available (blank and/or incomplete) were excluded, also opting for the exclusion of cases from rural areas, due to the inaccuracy of location of geographic coordinates in these areas.

After georeferencing, the leprosy cases were spatially distributed through the digital mesh of urban HDU, made available at the Social Vulnerability Atlas of the Institute for Applied Economic Research (*Atlas da Vulnerabilidade Social do Instituto de Pesquisa Econômica Aplicada* – IVS – IPEA)^21^.

In order to verify the places with the highest occurrence of leprosy in children under 15 years of age with disabilities (higher density of cases), according to the HDU, the Kernel density estimate was used, which consists of an exploratory interpolation method based on the definition of circular areas around points of occurrence of a phenomenon (cases), so that it generates a density surface for the identification of clusters^22^.

It is noteworthy that the search radius (bandwidth) used was the standard of the Esri® ArcGis™ Desktop 10.8 software, based on the Silverman bandwidth estimation formula, a non-parametric measure to estimate the probability density function of a random variable^22^.

In order to identify clusters in space and space-time, the sweep statistics technique was applied^23^ whose hypotheses are: H_0_: there are no clusters of leprosy cases in the HDU; H_1_: there are clusters of leprosy cases in the HDU.

With a view to identifying purely spatial clusters^23^, in which the distribution is heterogeneous and events are rare in relation to the general population, the discrete Poisson model was considered; no geographic overlapping of clusters; clusters with a maximum size equal to 50% of the exposed population; cluster with circular shape and 999 Monte Carlo replications. In addition, the spatial scanning technique was processed by controlling the occurrence of leprosy cases in children under 15 years of age by the size of the HDU population (population of children under 15 years of age) and attempts to detect clusters of high and low relative risks (RR). Type I error set at less than 5% was adopted as statistically significant (p<0.05).

For the space-time scan^23^, the same parameters mentioned above were used, and the maximum size of the temporal cluster equal to 50% of the study period was added, with precision in years, in the period from 2008 to 2018.

Finally, the analysis of spatial variations in temporal trends (SVTT) was carried out, with the aim of detecting geographic regions that present differences in relation to their temporal trends. The method uses the period in a fixed way, which, in this case, was considered the year unit, and gradually searches, through spatial windows of different sizes, the temporal trend inside and outside each one of these, having as hypotheses: H_0_, there are no differences between the temporal trends inside and outside the spatial windows; H_1_, time trends are different inside and outside the spatial windows^24,25^.

The SVTT analysis was performed considering a linear temporal trend from Poisson distribution: **Y_ij_~P_0_(E_ij_ × θ_ij_)**, where **Y_ij_** and **E_ij_** are, respectively, the observed and expected number of cases or events in a given area i and a period j, and θ_ij_ the relative risk in area i and period j^25^.

For the model in question, the expected number of cases in each area was calculated using indirect standardization: $E_{c}=p\times \frac{C}{P}$ , where **E_c_** is the expected number of cases within the window under H_0_; p is the population at the place of interest; C and P are the total number of cases and inhabitants, respectively.

The temporal trend was estimated using Poisson regression, considering time (in years) as an independent variable. Based on the count of occurrences of leprosy in children under 15 years of age, a maximum likelihood calculation was performed on all possible analysis windows. This maximum likelihood was compared with that of a large set of random data, derived from a Monte Carlo simulation, enabling statistical inference in the analyses^25^.

It is important to highlight that the clusters identified in these analyses do not indicate that the number of cases is high or low in relation to the rest of the HDU, but only identifies their temporal trend in comparison to the rest of the study units^25^.

The following parameters for the SVTT analysis were specified: type I error set at less than 5% (p<0.05), maximum spatial window size of the risk area of 50% of the study population; circular shape of the spatial window; maximum of 999 replications in Monte Carlo simulation; no geographic overlapping to report hierarchical clusters. Choosing the maximum size of the geographic cluster for 50% of the population was the usual recommendation for this type of analysis.

It should be noted that, for the spatial, space-time, and VETT analysis, the population of each HDU in Cuiabá (population of children under 15 years of age) was considered as the reference population (denominator) according to the last Demographic Census^17^.

For the analyses, the SaTScan software, version 9, was used, with choropleth maps (maps prepared with quantitative data using colors to establish a relationship between what is shown on the map and the caption), through the software Esri® ArcGis™ Desktop 10.8 and QGIS 3.14.

## RESULTS

Between 2008 and 2018, there were variations in the detection rates of leprosy in children under 15 years of age in Cuiabá, with rates ranging from one to eight cases per 100,000 inhabitants in the period.

With the exclusion of duplicate notifications, 514 cases of leprosy were reported in children under 15 years of age in Cuiabá. Most cases were females (50.9%), mostly MB (52.5%), with dimorphic clinical form (46.3%). As for DG, 52 cases (10.1%) had DG 1, 12 (2.3%) had DG 2, and 148 cases (28.8%) were not evaluated at the time of diagnosis. Table 1 brings the main characteristics of the cases in the investigated period.

**Table 1.** Clinical and social epidemiological characteristics of cases diagnosed with leprosy in children under 15 years of age, in an endemic municipality in the Brazilian Center-West (2008–2018).

	Characteristics	Frequency (n=514)	%
Gender	Male	252	49.1
	Female	262	50.9
Operational classification	Paucibacillary	244	47.5
	Multibacillary	270	52.5
Clinical form	Undetermined	104	20.2
	Tuberculoid	144	28.0
	Dimorphous	238	46.3
	Virchowian	22	4.3
	Unclassified/incomplete	6	1.2
Disability grading	Grade 0	302	58.8
	Grade 1	52	10.1
	Grade 2	12	2.3
	Not rated	148	28.8

For the georeferencing stage of the cases, from the total (514) three notifications whose addresses belonged to the rural area of Cuiabá and three that had a blank and/or incomplete address were excluded, making a total of 508 remaining cases. Of the 508 cases, 462 were georeferenced, with a total of 90.9% of georeferencing.

**Figure 1.**
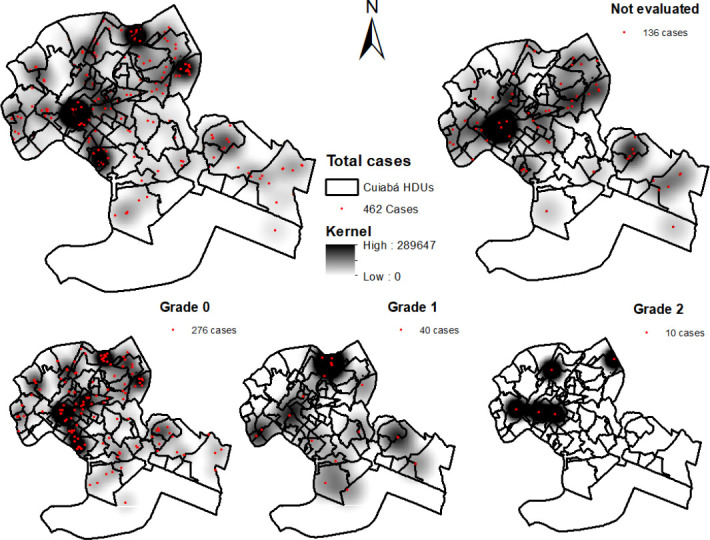
Spatial distribution and density of leprosy cases in children under 15 years old according to the Human Development Unit, Cuiabá (MT), Brazil, 2008–2018.

Figure 1 shows the spatial distribution of the density of leprosy cases in children under 15 years of age in the HDU of Cuiabá, where it was found that the highest densities of cases are found in the northeast region of the municipality. Regarding cases with DG 0 (276 cases), the highest density of cases was identified in the central, north, and south areas of the municipality, while for cases with DG 1 (40 cases), the areas with the highest density were identified in the north and south, while the highest densities of DG 2 (10 cases) were located in the north region. Finally, regarding the cases not assessed for DG at the time of diagnosis (136 cases), a higher density of cases was found in the north, central, and south areas.

HDU: human development unit.

Using purely spatial scanning, four risk clusters were identified, cluster 1 with RR=15.71 (95%CI 11.90–20.74), cluster 2 with RR=2.47 (95%CI 2.03–3.00), cluster 3 with RR=8.28 (95%CI 4.43–14.56), and cluster 4 with RR=2.19 (95%CI 1.64–2.91).

With the space-time scan, the conformation of three high-risk clusters can be observed, being cluster 1 with RR=36.08 (95%CI 24.14–53.92) in the period between 2015 and 2016, cluster 2 with RR=3.36 (95%CI 2.55–4.42) in the period between 2014 and 2016, and cluster 3 with RR=87.74 (95%CI 32.91–233.87) in 2012.

Finally, with the application of SVTT, a cluster with an internal temporal trend of -30.86%/year and an external temporal trend of -2.28%/year was identified. The internal temporal trend represents the degree of growth or reduction in the number of leprosy cases over the years within the cluster, being compared with the external trend, that is, the trend throughout the city of Cuiabá not belonging to the same cluster. It was possible to observe that, in the identified cluster, the internal trend of reduction was more intense than the external trend, expressing a greater reduction in the number of leprosy cases, compared to the rest of the municipality.

Table 2 shows the clusters identified through purely spatial, spatio-temporal, and SVTT scanning techniques.

**Table 2.** Results of spatial scanning, spatiotemporal, and spatial variations in temporal trends to identify problematic territories for the occurrence of childhood leprosy and its vulnerabilities in the Center-West of Brazil.

Analysis	Cluster	Number of cases	Number of HDU	Population	Period	p-value	Relative risk	(95%) Confidence interval
Spatial	1	56	1	4,021	2008–2018	<0.01	15.71	11.90–20.74
	2	155	32	78,418	2008–2018	<0.01	2.47	2.03–3.00
	3	10	1	1,232	2008–2018	<0.01	8.28	4.43–15.46
	4	54	10	26,366	2008–2018	<0.01	2.19	1.64–2.91
spatiotemporal	1	25	1	4,021	2015–2016	<0.01	36.08	24.14–53.92
	2	58	29	69,416	2014–2016	<0.01	3.36	2.55–4.42
	3	4	1	505	2012–2012	<0.01	87.74	32.91–233.87
SVTT	1	22	6	12,962	2008–2018	0.03	-30.869%*	-2.281%^†^

* Cluster internal time trend; ^†^Cluster external time trend. SVTT: spatial variation in time trends; HDU: human development unit.

**Figure 2.**
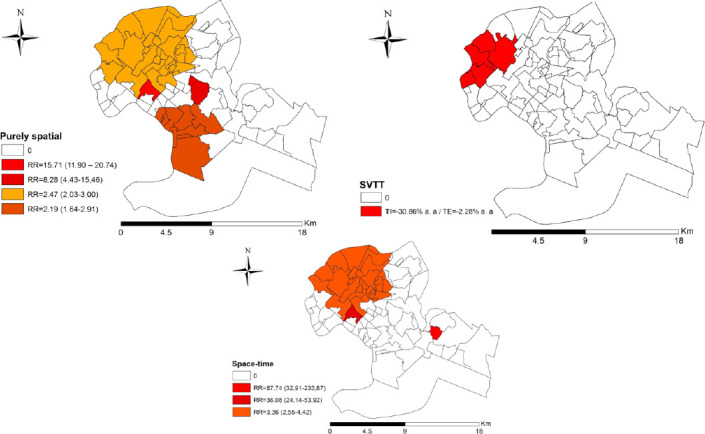
Spatial and spatiotemporal scan, and spatial variations in temporal trends of leprosy cases in children under 15 years of age in Cuiabá (MT), Brazil, 2008–2018.

In Figure 2, it is evident that, with the results of applying the purely spatial scan, the cluster with the highest RR was located in the west and east regions (15.71), followed by a cluster located in portions of the west and north regions (8.28), with the cluster with the lowest RR located in the north and south regions (2.47 and 2.19) of the city. The same figure also shows the clusters identified through the space-time scan, with the cluster with the highest RR located in the southern region (87.74), followed by a cluster located in the western region (composed of a HDU with 36.08), and the cluster with the lowest RR located in the north and west regions (3.36). Finally, with the application of SVTT, a cluster located in the western region of Cuiabá was identified.

SVTT: spatial variations in temporal trends.

## DISCUSSION

In Cuiabá, the spatial distribution of leprosy in children under 15 years of age is heterogeneous, with regions that have a higher concentration of cases and clusters of risk. Leprosy cases with physical disabilities were distributed across practically the entire urban area of Cuiabá, while the highest risk clusters were located in regions characterized by low socioeconomic indicators. The spatial analysis indicated the regions with the highest occurrence of leprosy in Cuiabá, bringing evidence that the municipality faces operational difficulties in controlling the disease, especially in early diagnosis.

Leprosy in children under 15 years of age in Cuiabá classifies the municipality as a scenario of very high endemicity for the disease, being one of the Brazilian cities that most notified the disease in the period from 2008 to 2018^26^.

Most cases were classified as MB and with a dimorphic clinical form. Studies indicate that people affected by the MB forms of the disease are more likely to develop physical disabilities, especially the involvement of hands and feet, impairing self-care and the functionality of the affected limb^3,27^_._

As for DG, in Cuiabá, 10.1% had DG 1; and 2.3% had DG 2 at the time of diagnosis, so that the percentage of disabilities was 12.4%. Comparing with Brazil, in the period from 2009 to 2018, the observed proportion of DG 1 was 3.1%, whereas, for DG 2, the observed proportion was 2.7%^28^. These epidemiological data confirm that Cuiabá has a percentage of DG higher than the national average, especially DG 1, suggesting deficiencies in the early diagnosis of leprosy in the population studied^28^.

The proportion of new cases of leprosy diagnosed with DG, especially DG 2, is an important indicator of late diagnosis, especially in children under 15 years of age, indicating that this population, from an early age, already has contact with *Mycobacterium leprae* ^27,28^.

It is also noteworthy that a large portion did not present disabilities at the time of diagnosis (58.8%), but 30.7% were not evaluated, results that suggest fragility in health services as well as in the performance and qualification of health professionals, which may be linked to the management of the disease, and non-existence or non-adherence to protocols.

With the Kernel density estimator, it was possible to verify that the cases of leprosy with disabilities were distributed in practically the entire urban area of Cuiabá, however, areas with a higher concentration of cases were identified in the north and west administrative regions, followed by the east region.

Through spatial scanning, the highest RR were identified in the east and west regions with 15.71 and 8.28, respectively. It is worth mentioning that, in these regions, income varies between low, medium-low, and medium, with the fact that the highest rates related to demographic density are also found in these regions, suggesting a relationship between the occurrence of leprosy in children under 15 years of age and social vulnerability^29,30^.

Social vulnerabilities are an important aspect in leprosy, and the difficulty of accessing health services, the gaps in knowledge about the disease, often explained by the low quality and time of education/literacy, and the participation of people in society strongly generate the maintenance of the disease chain^29^. Malnutrition and incomplete immunization show risks for acquiring the disease^30^.

With the space-time scan, three clusters of risk for leprosy were identified, involving the years 2012 to 2016. In Cuiabá, in 2015, local health services were mobilized to carry out actions related to active search for cases, focusing on schoolchildren aged 5 to 14 years, with campaigns to examine and treat this population and their possible contacts^31,32^. The aforementioned campaign may have had an impact on the studied region, however, during the previous or subsequent years, no other strategy linked to the active detection of leprosy was found in public archives.

Based on the SVTT analysis, a cluster with an internal temporal trend of -30.86%/year and an external temporal trend of -2.28%/year was identified, located in the western region of Cuiabá. This result indicates that, within the agglomeration, the internal trend of reduction was more intense than the external trend, expressing a greater reduction in the number of leprosy cases, compared to the rest of the municipality.

It is noteworthy that the western region of Cuiabá has the lowest number of family health units in the municipality, so it is questionable whether the active search for cases is being carried out effectively, as well as whether this decreasing temporal trend is real or rather a reflection of underreporting/underdiagnosis of cases.

One can hypothesize that the municipality faces difficulties in the early diagnosis of the disease and, consequently, to prevent its continuous transmission. Studies carried out with leprosy cases in the same age group have already identified that early exposure to the pathogen led to a late diagnosis^33,34^.

The fact that there is DG 2 in children and adolescents is worrying, showing that it is far below the goal of reaching zero status of the disease^35,36^. Leprosy is highly disabling when not properly treated, especially in this population, which can influence school performance and cause problems related to social limitations, discrimination, self-esteem, and stigma^37^. Classically, the presence of the disease is also related to stigma, especially when deformities occur^1^. A comprehensive assessment of children under 15 years of age needs to be increased within the scope of Primary Health Care (PHC)^38^.

Effective actions to overcome leprosy involve more investments in health and the development of different intervention strategies in the community, both in research and surveillance in the community or critical territories and in the provision of individual care to children, adolescents and their families^39^. Education and communication in health with the community is fundamental for the mobilization, awareness, and empowerment of people, especially in the age group of children under 15 and adolescents^40-42^.

An ally in the fight against leprosy is chemoprophylaxis. According to World Health Organization (WHO) guidelines, single-dose rifampicin (SDR) as post-exposure prophylaxis (PEP) can be used in children and adolescents^36^. SDR-PEP can be studied by policy makers and/or health managers based on WHO recommendations and effectiveness and feasibility^36,43,44^.

One of the limitations in this study was related to the database used, with secondary data, which may contain inconsistent information regarding quantity and quality, with the presence of ignored and/or incomplete data. Another limitation involves georeferencing, which allows identifying a certain number of locations in urban areas and is not accurate for rural areas. Finally, it should be noted that the analyses were processed by year, so that oscillations in case detection considering smaller units (months, days) could not be captured.

As a conclusion of the study, in Cuiabá, through the spatial analysis used, it was identified that leprosy in children under 15 years of age presents heterogeneous spatial behavior, located mainly in territories with social vulnerability and lack of health services. Based on the present study, a critical situation of leprosy in children under 15 years of age and the occurrence of disabilities can be observed, suggesting that the disease in the analyzed scenario constitutes a public health problem still far from being solved.
